# Traditional Strategies vs. Social Media for Recruitment of Older Adults in a Longitudinal Study

**DOI:** 10.1093/geroni/igaf122.584

**Published:** 2025-12-31

**Authors:** Kelley Jackson, David Newman, Alexandru Pasarariu, KwangSoo Yang, Ruth Tappen

**Affiliations:** Florida Atlantic University, Boca Raton, Florida, United States; Florida Atlantic University, Boca Raton, Florida, United States; Florida Atlantic University, Boca Raton, Florida, United States; Florida Atlantic University, Boca Raton, Florida, United States; Florida Atlantic University, Boca Raton, Florida, United States

## Abstract

Over time, a variety of traditional recruitment strategies were employed in a 5-year longitudinal study of cognition and driving behavior in older adults. Eligible participants were older (≥65) adult drivers residing in South Florida in possession of a valid driver’s license and insurance who were willing to participate in quarterly cognitive testing and to allow sensors, including driver-facing video, forward-facing video, GPS, and telemetry to capture their driving behavior, to be installed in their personal vehicles. Initial recruitment strategies included flyer distribution (virtually and onsite), health fairs, personal referrals from existing participants, and presentations to community groups. In the fourth year of our study, a digital marketing consultant was engaged to assist the research team in launching geographically targeted, customized ads appealing to older adults meeting inclusion criteria via Facebook and Instagram platforms. Close to 800 individuals responded, indicating their interest electronically, and were contacted by research team members over the next 9 months while traditional recruitment strategies continued. Those who actually enrolled in the study during this time period via social media (n = 57) when compared to traditional strategies (n = 28) were younger t(83) =2.8, p=.003, and had higher MoCA scores t(83) =2.8, p=.003. The two groups of enrolled participants did not differ in terms of gender (p=.191), ethnicity (p=.326), race (p=.236), or years of education (p=.167). In conclusion, social media shows promise to be an effective stand-alone or adjunctive strategy for the recruitment of older adults for relatively complex research studies.

